# Patient-Reported Outcome Measures (PROMs) and Frailty Assessments Before and After Transcatheter Aortic Valve Implantation (TAVI): A Review of Current Evidence

**DOI:** 10.7759/cureus.106060

**Published:** 2026-03-29

**Authors:** Adeogo Olusan, Enays Amrani, Abdulrahman Kolapo, Iyare Nehikhare

**Affiliations:** 1 Cardiology, University Hospitals of Leicester National Health Service (NHS) Trust, Leicester, GBR

**Keywords:** aortic stenosis (as), frailty assessment, geriatric cardiology, patient reported outcome measures, physical performance, quality of life (qol), surgical risk calculator, transcatheter aortic valve intervention (tavi)

## Abstract

Transcatheter aortic valve implantation (TAVI) has become a paradigm shift in the treatment of elderly and frail patients with severe aortic stenosis, offering a minimally invasive alternative to conventional surgical aortic valve replacement. As the indications for TAVI expand to include lower-risk patients, there is a growing need for sophisticated patient assessment methodologies that capture the complex interplay between physical and functional status, quality of life, and treatment outcomes. This review aims to synthesize the current evidence on the use of patient-reported outcome measures (PROMs) and frailty assessments before and after TAVI, with a focus on their utility in predicting treatment outcomes and improving patient-centered care. A comprehensive literature search was conducted to identify studies published in peer-reviewed journals that investigated the use of PROMs and frailty assessments in the context of TAVI. The review found that PROMs and frailty assessments are increasingly being used to evaluate the pre- and post-procedural status of patients undergoing TAVI. These assessments have been shown to predict treatment outcomes, including mortality, morbidity, and quality of life, and to inform treatment decisions. The review also highlights the importance of integrating PROMs and frailty assessments into routine clinical practice to optimize patient outcomes and improve patient-centered care. In conclusion, this review demonstrates the growing body of evidence supporting the use of PROMs and frailty assessments in the context of TAVI. By incorporating these assessments into routine clinical practice, healthcare providers can better identify patients at risk of poor outcomes, optimize treatment strategies, and improve patient-centered care. Future research should focus on developing and validating PROMs and frailty assessments that are specific to the TAVI population and on exploring the impact of these assessments on treatment outcomes and healthcare utilization.

## Introduction and background

TAVI as a treatment paradigm

Transcatheter aortic valve implantation (TAVI) has emerged as the preferred treatment approach for elderly and frail patients with severe aortic stenosis deemed unsuitable for conventional surgical aortic valve replacement [1]. The widespread expansion of TAVI indications from high-risk patients to lower-risk cohorts necessitates increasingly sophisticated patient assessment methodologies that extend beyond traditional mortality risk scores [2]. Contemporary clinical practice recognizes that operative risk stratification must incorporate comprehensive evaluation of patient vulnerabilities, including frailty status and anticipated functional recovery, alongside objective clinical parameters [3]. This integration of geriatric principles into TAVI patient assessment represents a paradigm shift towards truly patient-centered care, where quality of life and functional independence assume equal importance to survival metrics [4].

The aging population's expanding prevalence of aortic stenosis creates substantial epidemiological challenges for healthcare systems globally [5]. While TAVI procedures have demonstrated substantial survival benefits and symptom relief in most patients, a significant proportion fail to experience meaningful functional improvement or quality of life gains, particularly those with advanced frailty or multiple comorbidities [6]. This heterogeneity in treatment outcomes underscores the critical importance of developing robust preoperative assessment strategies that can accurately predict which patients will derive genuine benefit from intervention [7]. Patient-reported outcome measures (PROMs) and standardized frailty assessments have emerged as essential tools for capturing the complex physiological and psychosocial dimensions that determine success in TAVI candidates [8].

## Review

Patient-reported outcome measures (PROMs) in TAVI: assessment tools and applications

The Kansas City Cardiomyopathy Questionnaire (KCCQ) as Primary PROM

The Kansas City Cardiomyopathy Questionnaire (KCCQ-12) has become the predominant patient-reported outcome measure for TAVI populations, offering superior advantages over both the traditional New York Heart Association (NYHA) functional classification and longer generic health questionnaires [9]. In a contemporary cohort of 366 TAVI patients, baseline KCCQ-12 scores demonstrated exceptional utility in stratifying patients into distinct functional improvement trajectories, with failure to improve defined as scores remaining below 60 or changing less than 10 points at one year [9]. These improvements in KCCQ scores were strongly associated with clinically meaningful changes in patients' perceived quality of life, with particularly robust effects observed in the physical function and clinical summary domains.

The predictive capacity of KCCQ extends beyond simple post-procedural symptom assessment. Multivariate analysis incorporating baseline KCCQ overall scores alongside comorbidity burden and frailty status revealed that the KCCQ independently predicted functional improvement outcomes, with adjusted odds ratios of 0.3 for each 10-point decrement in baseline scores (p=0.04) [9]. When KCCQ assessments are paired with objective performance measures such as the 6-minute walking test (6MWT), clinicians obtain a comprehensive picture of functional capacity that encompasses both self-perception and objective functional reserve [10]. In a population of 88 TAVI patients with severe aortic stenosis, approximately 50% demonstrated clinically significant improvements in the physical function domain of SF-36 (≥15 points) at 12 months, with stronger baseline impairment predicting a greater likelihood of meaningful improvement.

EuroQol and Short-Form Health Measures

The EuroQol-5-Dimension questionnaire (EQ-5D, including the visual analogue scale variant EQ-5D-VAS) provides a complementary assessment of health utility and quality of life in TAVI populations [11]. A TAVI-specific predictive model incorporating the EQ-5D-VAS alongside the Clinical Frailty Scale and American Society of Anesthesiologists (ASA) scores achieved superior predictive accuracy for one-year mortality compared to traditional EuroSCORE II (AUC 0.800 vs. 0.659, p = 0.002), emphasizing the incremental value of patient-reported health perception metrics. The short form (SF-36/SF-12) questionnaires similarly demonstrate sensitivity to TAVI-related improvements, with meta-analytic evidence showing significantly greater short-term improvements in SF physical summary scores at one-month post-TAVI compared to surgical aortic valve replacement, though differences attenuate by one-year follow-up [12].

Shared decision-making frameworks increasingly incorporate multi-dimensional quality of life assessments alongside clinical data, as they provide essential information about patient priorities and values that inform realistic expectations and treatment planning [13]. In addition to comprehensive health status measurement, the SF-36 and similar tools capture emotional well-being dimensions, mental summary scores, for instance, that reflect the psychosocial impact of severe aortic stenosis and its treatment. The combination of disease-specific measures (KCCQ) with generic health status tools (SF-36, EQ-5D) provides a balanced assessment approach that satisfies both clinical utility and research comparability requirements [9].

Experience Measures and Patient Satisfaction

Beyond traditional outcome metrics, patient-reported experience measures (PREMs) capture satisfaction with care delivery processes and patient engagement throughout the TAVI pathway. A multidisciplinary TAVI program incorporating nurse-coordinated comprehensive assessment and follow-up demonstrated significantly higher patient satisfaction scores compared to standard care (9.8 vs. 8.9 on 10-point scales), with associated reductions in emergency department visits (11.8% vs. 31.3%) and readmissions (1.2% vs. 23.4%) [14]. The VALVEX study similarly documented that 96% of TAVI patients would recommend the procedure to others, with the highest satisfaction ratings for information provision and nursing support, though delays in diagnosis-to-treatment intervals and patient involvement in decision-making processes remained identified improvement areas [8].

Novel approaches to informed consent utilization of comic-based educational materials (versus conventional text-based consent) significantly enhanced patient satisfaction and reduced pre-procedural anxiety compared to standard informed consent processes [15]. Patients receiving comic-based informed consent reported higher satisfaction (29 vs. 25 points on the Client Satisfaction Questionnaire, p<0.001), lower anxiety (34 vs. 39 points on Spielberger scales, p<0.001), and 61% versus 30% reported feeling "relaxed" prior to the procedure (p<0.001), demonstrating that PREMs serve as sensitive indicators of care quality and patient experience. These experience measures, while distinct from clinical outcomes, correlate with improved compliance, rehabilitation participation, and ultimately functional recovery post-TAVI [8].

Frailty assessment methodologies and comparative validation

**Figure 1:**
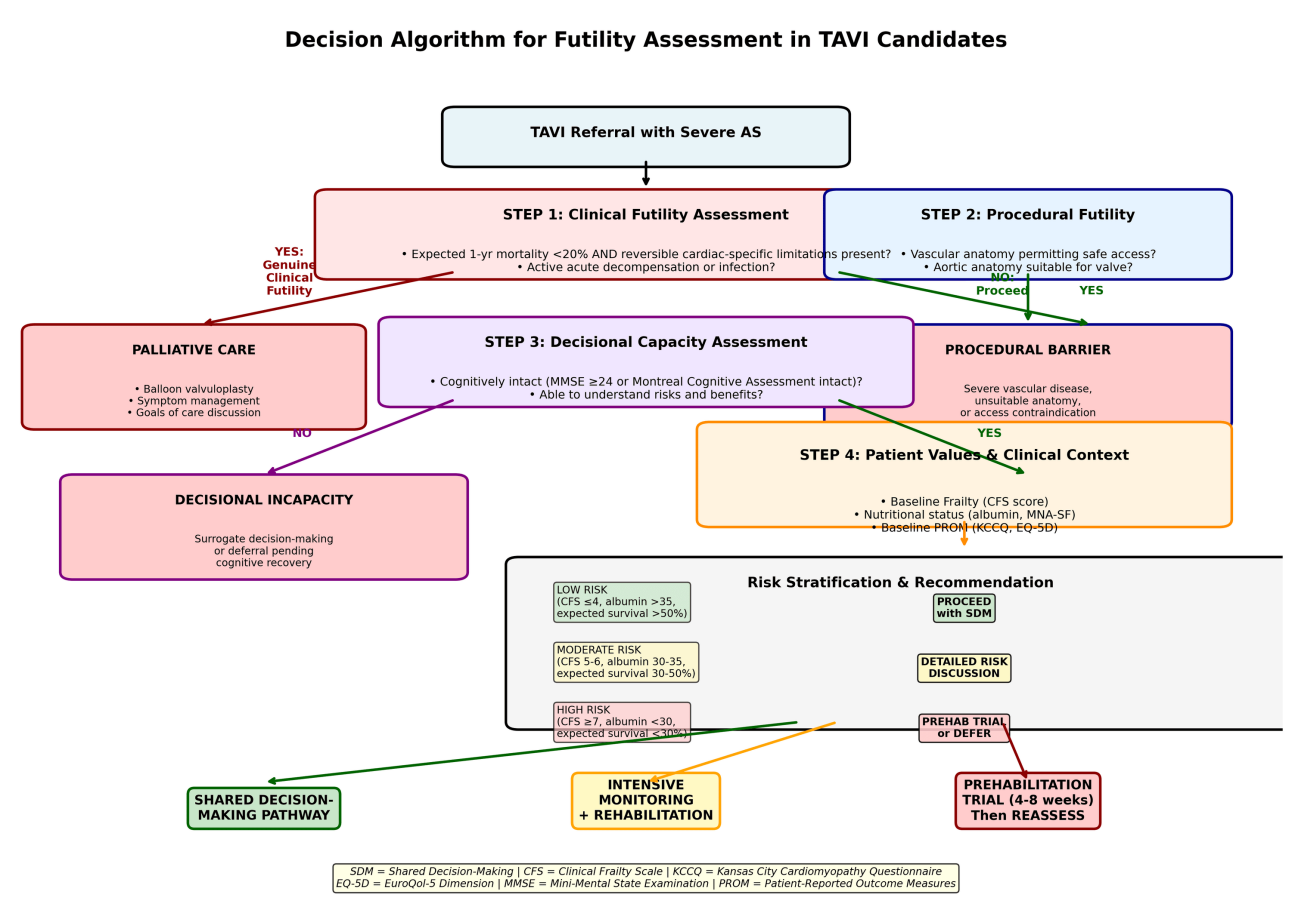
Decision algorithm for futility assessment in TAVI candidates

Although the studies included in this review are heterogeneous, the authors stratify the evidence by quality and design type as follows (Table 1, Figure 1): These include large prospective registry studies (n>500) with multi-center validation, moderate-sized single-center cohort studies (n=200-500) with specific clinical populations, and smaller feasibility and pilot studies (n<100).

**Table 1:** Frailty tool stratification by evidence quality, clinical setting, and time requirements

Frailty Assessment Tool	Evidence Quality Tier	Administration Time	Clinical Setting	Predictive Validity for TAVI	Recommended Implementation
Clinical Frailty Scale (CFS)	HIGH (n>2,000)	5-10 min	Rapid screening (ED, clinic)	Excellent (AUC 0.69-0.80)	First-line screening for ALL TAVI candidates
Comprehensive Geriatric Assessment (CGA) [23]	GOLD STANDARD	30-45 min	Specialist evaluation	Excellent (AUC 0.88-0.92)	High-risk patients (CFS ≥5 or specific concerns)
Fried Frailty Phenotype	HIGH (n>1,000)	15-20 min	Research/detailed assessment	Good (AUC 0.71)	Research and validation studies
Short Physical Performance Battery (SPPB)	MODERATE (n=200-500)	10-15 min	Comprehensive assessment	Good (predicts disability)	Secondary assessment when CFS ≥5
Simple FRAIL Questionnaire	MODERATE (n=200-400)	2-3 min	Quick office screening	Good (sensitivity 88%)	Rapid telephone or clinic screening
Timed Up-and-Go (TUG)	MODERATE (n=100-300)	5 min	Mobility screening	Moderate (AUC 0.60-0.70)	Component of CGA or mobility assessment
Kihon Checklist (KCL)	MODERATE (n=986)	10-15 min	Broader assessment	Good (independent predictor)	When comprehensive clinic assessment desired
Essential Frailty Toolset (EFT)	LOW (n<200)	15 min	Detailed evaluation	Good (excellent discrimination)	High-risk or complex patients
Frailty Index (FI)	HIGH (n>1,000)	20-30 min	Research/epidemiology	Very Good (AUC 0.75-0.80)	Population-level research
PRISMA-7 Questionnaire	MODERATE (n=200-300)	3-5 min	Primary care screening	Moderate (sensitivity 76%)	Primary care pre-referral screening

Clinical Frailty Scale and Its Prognostic Utility

The Clinical Frailty Scale (CFS), a brief 9-point assessment synthesizing clinical judgment with components of comorbidity, cognitive impairment, and disability, has emerged as the most widely adopted frailty screening tool for TAVI populations [16]. Among 377 TAVI candidates with aortic stenosis, frailty prevalence ranged substantially according to assessment methodology, 17.6% via Fried criteria but 49.8% when utilizing the CFS, reflecting the tool's sensitivity to subtle functional decline and dependency in instrumental activities of daily living. When compared with the Fried Frailty Phenotype and comprehensive geriatric assessment-based frailty indices, the CFS demonstrated superior predictive capacity for the composite outcome of one-year mortality or readmission, particularly in identifying disability for instrumental activities of daily living.

The CFS has demonstrated consistent associations with unfavorable clinical outcomes across multiple TAVI studies. A meta-analysis of frailty assessment tools in aortic valve interventions confirmed that CFS scores independently predicted 12-month mortality risk following either TAVI or surgical valve replacement, supporting its routine use in preoperative patient assessment [17]. Furthermore, the CFS's brevity and feasibility for rapid bedside assessment make it particularly suited for acute care settings and pre-procedural triage, where time constraints limit comprehensive geriatric evaluation. In the SCOPE I trial cohort stratified by frailty, patients meeting frailty criteria (16.5% of 739 participants) demonstrated significantly elevated risk of unfavorable outcomes according to Valve Academic Research Consortium (VARC-3) criteria compared with non-frail peers throughout three-year follow-up [18].

Comprehensive Geriatric Assessment and Multidimensional Approaches

A Comprehensive Geriatric Assessment (CGA), a multidimensional evaluation encompassing functional independence (activities of daily living, instrumental ADL), cognitive status, mood (depression screening), nutritional status, mobility, and social support, represents the gold-standard methodology for frailty identification in older adults [19]. A novel geriatric assessment-based risk score derived from 562 TAVI candidates at tertiary centers demonstrated superior discrimination for one-year mortality or functional decline compared to conventional surgical risk models [2]. This GASS-TAVI score incorporated malnutrition risk (mini nutritional assessment-short form), basic ADL impairment, renal function (eGFR), and echocardiographic parameters (pulmonary artery systolic pressure), achieving an AUC of 0.92 in derivation and 0.87 in validation cohorts versus an AUC of 0.60 for the Society of Thoracic Surgeons score and 0.73 for the Multidimensional Prognostic Index.

Multiple specialized geriatric assessment tools have demonstrated efficacy in predicting TAVI outcomes beyond mortality. The Kihon Checklist (KCL), covering physical, cognitive, and social domains, identified frailty in 47.4% of 986 TAVI patients and independently predicted all-cause mortality (adjusted hazard ratio 1.771, 95% CI 1.096-2.862, p<0.02), with social isolation and physical function emerging as particularly strong risk factors [20]. The Essential Frailty Toolset (EFT), Short Physical Performance Battery (SPPB), and frailty index based on CGA (IF-VIG) each demonstrated stronger associations with adverse outcomes compared to the Fried criteria or FRAIL scale in a geriatric cardiology cohort [16]. These multidimensional assessments capture the complexity of physiological vulnerability in ways that single-domain tests cannot, making them particularly valuable for clinical decision-making in high-risk populations [1].

Physical Performance and Sarcopenia Markers

Lower extremity physical performance, measured via the short physical performance battery (SPPB) assessing balance, gait speed, and sit-to-stand capacity, provides objective quantification of physical frailty phenotype criteria [21]. Preoperative SPPB assessment in 92 consecutive TAVI patients (median age 85 years, 63% female) did not independently predict hospitalization-associated disability (defined as ≥5-point Barthel Index decline) when adjusted for baseline functional capacity and postoperative rehabilitation intensity. However, the SPPB remains valuable as a component of multidimensional frailty assessment; its predictive capacity may be insufficient as a standalone tool, necessitating complementary assessment of cognitive function, nutritional status, and baseline disability [21].

Sarcopenia, characterized by low muscle mass combined with reduced strength or physical function, intersects substantially with frailty phenotypes and independently predicts TAVI outcomes. Analysis of computed tomography-derived psoas muscle volume in 197 TAVI patients revealed that while psoas muscle area showed high specificity (84%) for frailty detection, sensitivity remained low (28%), limiting its utility as a standalone screening tool [22]. However, when sarcopenia was formally diagnosed using European Working Group on Sarcopenia in Older People (EWGSOP) criteria incorporating psoas volume index, handgrip strength, and timed up-and-go test, sarcopenia was independently associated with increased one-year mortality (log-rank test, p=0.026). These findings underscore the importance of multifaceted physical assessment in TAVI candidates, recognizing that single anthropometric markers inadequately capture musculoskeletal frailty [22].

Clinical Application: Tiered Approach

Tier 1 (Universal) includes CFS, albumin, and eGFR, and can be completed within 5-10 minutes across all settings. Tier 2 (High-risk) involves CGA, SPPB, cognitive screening, and nutritional assessment, typically requiring 30-45 minutes and specialist input. Tier 3 (Research) comprises advanced measures such as the Fried Phenotype, Frailty Index, and specialized biomarkers.

Comorbidities, specific geriatric markers, and predictive models

Nutritional Status as Core Frailty Component

Malnutrition and nutritional risk emerge as particularly strong independent predictors of poor TAVI outcomes, appearing across multiple validated prognostic models. In 100 TAVI patients with a mean age of 84±4 years, depression (Geriatric Depression Scale-15) and malnutrition independently predicted 24-month all-cause mortality in multivariable Cox models (HR 4.381, 95% CI 1.787-10.743, p=0.001 and HR 3.076, 95% CI 1.151-8.217, p=0.025, respectively), with combined screening for these geriatric markers substantially improving prognostic discrimination [24]. The GASS-TAVI study identified malnutrition risk (evaluated by MNA-SF score) as a key independent predictor of one-year mortality or functional decline [2].

A novel nutritional index, TCBI (triglycerides, total cholesterol, and body weight index), derived from readily available clinical laboratory values, demonstrated substantial associations with frailty markers and three-year mortality risk in 824 TAVI patients from the LAPLACE-TAVI registry [25]. Patients in the lowest TCBI tertile showed substantially impaired motor functions, reflecting frailty, and significantly elevated cumulative mortality incidence compared to other tertiles. Remarkably, the negative prognostic impact of low TCBI was amplified among patients experiencing serious pre-procedural complications, with a hazard ratio of 4.9 (95% CI 1.9-12.5, p<0.001) in this subgroup [25]. These findings underscore the urgency of nutritional assessment and optimization in TAVI candidates as an actionable, modifiable factor.

Renal Function and Multi-System Comorbidity

Severe chronic kidney disease and reduced renal function (eGFR) independently predict TAVI failure to improve and adverse outcomes. Among 366 TAVI patients, those experiencing failure to improve in functional status were significantly more likely to have severe CKD (13% vs. 2%), and multivariate analysis confirmed severe CKD as an independent predictor with an adjusted odds ratio of 5.7 (p=0.004) [9]. Similarly, in the GASS-TAVI cohort, lower eGFR independently predicted one-year mortality or functional decline [2]. A novel prediction model incorporating eGFR (estimated via cystatin C), N-terminal pro-brain natriuretic peptide (NTproBNP), and patient-reported measures (KCCQ, EQ-5D-5L) achieved substantially superior predictive accuracy for length of hospital stay exceeding six days compared to EuroSCORE II alone [26].

Baseline albumin level, a marker of nutritional status, hepatic synthetic function, and overall physiological reserve, demonstrates independent prognostic significance in multiple TAVI cohorts. Patients failing to improve post-TAVI had significantly lower baseline albumin (36 g/L vs. 38 g/L, p<0.05) [9], while severely frail patients assessed with the Kihon Checklist similarly showed lower albumin levels [20]. These composite markers of physiological vulnerability, encompassing renal, hepatic, and nutritional dimensions, provide complementary information to traditional cardiac risk stratification and support comprehensive multisystem assessment [19].

Cognitive Impairment and Mental Health

Mild cognitive disorder affects approximately 55% of TAVI candidates and substantially impacts post-procedural outcomes. In a secondary analysis of the TAVI-COMIC randomized trial among 199 TAVI patients, 54.8% demonstrated mild cognitive disorder (Montreal Cognitive Assessment score <26) [27]. While patients with and without mild cognitive disorder reported similar general health improvements post-TAVI, those with cognitive impairment demonstrated significantly reduced functional independence, with 54% less frequent ability to perform procuration, 55% less frequent walking ability without difficulties, and 52% less frequent functional independence (all p<0.05). Additionally, patients with mild cognitive disorder experienced more frequent postoperative delirium (8.3% vs. 1.1%, p=0.049) [27].

Depression screens using the Geriatric Depression Scale (GDS-15) and broader cognitive assessment with the Montreal Cognitive Assessment (MoCA) or Mini-Mental State Examination (MMSE) should constitute standard components of comprehensive TAVI patient evaluation. In the CAPITA study examining cognitive outcomes in 148 TAVI patients (mean age 80.5 years, 43% female), global cognitive functioning improved significantly from baseline to three-month follow-up (z-score 0.02±0.52 to 0.15±0.48, p<0.001), with particular improvements in memory and language domains [28]. Notably, patients with the worst baseline cognitive functioning demonstrated the largest improvements in cognitive function (0.30±0.55 SD improvement in the lowest tertile vs. 0.09±0.27 and 0.01±0.16 in the intermediate and highest tertiles, p=0.002).

Integrated risk stratification models and multi-domain assessment

Developing TAVI-Specific Predictive Models

Traditional cardiac risk scores such as EuroSCORE II demonstrate limited discrimination in TAVI populations, with area under the curve values of 0.597-0.660 in various cohorts, substantially lower than contemporary TAVI-specific models [26]. A machine learning approach incorporating simple clinical variables (ASA score and Clinical Frailty Scale) achieved superior one-year mortality prediction (AUC 0.800) compared to EuroSCORE II (AUC 0.659, p=0.002) in 284 TAVI candidates at a single German center [11]. Translating these results into practical clinical decision-support tools, a decision tree algorithm identified risk cut-offs for one-year mortality: <0.02 for low risk, 0.02-0.15 for intermediate risk, and >0.15 for high risk.

Incorporating objective performance measures alongside subjective clinical assessment further enhances predictive models. A novel Linear Prediction Score (LPS1) combining eGFR, NTproBNP, and KCCQ achieved an AUC of 0.761 for mortality prediction in 169 TAVI patients, significantly exceeding EuroSCORE II performance (AUC 0.597, p=0.035) [26]. For predicting prolonged length of hospital stay (>6 days), a second model (LPS2) incorporating NTproBNP, eGFR, and EQ-5D-5L values achieved an AUC 0.677 (p < 0.001), identifying modifiable factors that contribute to perioperative morbidity. These personalized risk models enable precise patient counseling regarding anticipated hospital stays, resource utilization, and recovery timelines [11].

Postoperative Functional Trajectories and Disability Prevention

Understanding anticipated functional trajectories post-TAVI informs realistic goal setting and identifies patients requiring intensive postoperative rehabilitation. Among 88 TAVI patients with preserved ejection fraction and severe aortic stenosis, clinically significant improvement (≥15-point increase) in the SF-36 physical function domain at 12 months was observed in approximately 50% of patients, with stronger baseline impairment predicting a greater likelihood of improvement [10]. Remarkably, no significant associations were identified between improvement in physical function scores and routine baseline clinical parameters, suggesting that patient-specific factors beyond standard risk assessment determine recovery trajectory.

A proposed threshold of 15-point improvement in physical function and physical role domains of SF-36 provides clinically meaningful benchmarking for TAVI outcomes [10]. Hospitalization-associated disability (HAD), defined as a ≥5-point decline in the Barthel Index from the preoperative baseline to discharge, occurred in 26.1% of TAVI patients despite aggressive postoperative rehabilitation, with older age and comorbidity burden increasing risk [21]. These findings emphasize that achievement of procedural success alone (hemodynamic improvement, absence of acute complications) does not guarantee functional recovery; intensive, personalized rehabilitation initiated from postoperative day one represents essential adjunct therapy.

Disease-Specific and Contextual Outcomes Beyond Mortality

The Valve Academic Research Consortium (VARC)-3 composite outcome, integrating freedom from all-cause mortality, freedom from all strokes, freedom from hospitalization for valve-related causes, and sustained improvement in KCCQ score, provides a disease-specific endpoint definition more relevant to patient-centered care than mortality alone [18]. In the SCOPE I trial of 739 TAVI patients stratified by frailty, while both groups achieved similar improvements in patient-reported health status measures, frail patients demonstrated significantly elevated risk of VARC-3 unfavorable outcomes (risk ratio 1.38, 95% CI unspecified) throughout the three-year follow-up. This divergence between symptomatic improvement and objective complication rates highlights the importance of comprehensive outcome assessment incorporating both patient perception and clinical events [18].

Clinical implementation and optimization strategies

Multidisciplinary Team Assessment and Frailty Management

Comprehensive TAVI programs incorporating specialized frailty response protocols and multidisciplinary team involvement yield substantially improved patient outcomes compared to standard care. A novel Frailty Response Program for TAVI candidates with aortic stenosis incorporates implementation strategy and clinical protocol focused on malnutrition identification, patient education, general practitioner notification, comprehensive geriatric assessment, and cardiac rehabilitation integration [29]. While outcomes remain pending from ongoing randomized controlled trial evaluation, protocol components address the multidomain nature of frailty and facilitate coordinated prehabilitation.

Multidisciplinary programs coordinated by specialized TAVI nurses demonstrate feasibility and significant benefits for patient-centered outcomes. In 154 TAVI patients (87 in the nurse-coordinated program vs. 67 in standard care), the nurse program group showed significantly better functional class achievement, fewer emergency department visits (11.8% vs. 31.3%), and substantially lower readmission rates (1.2% vs. 23.4%) [14]. Overall patient satisfaction with the entire TAVI process was significantly higher in the nurse program cohort (9.8 vs. 8.9 on a 10-point scale, p < 0.05), with the greatest satisfaction improvements regarding information received and interpersonal care interactions. These structural improvements in care delivery directly translate to reduced healthcare utilization and enhanced functional recovery.

Preoperative Prehabilitation and Rehabilitation Pathways

Cardiac rehabilitation protocols incorporating structured exercise (endurance and strength training), nutritional counseling, occupational therapy, and psychological support offer potential for improving outcomes in frail TAVI candidates [30]. Rehabilitation program components should address the multidomain nature of frailty: (i) physical training targeting muscle mass, strength, balance, and coordination; (ii) nutritional optimization and counseling; (iii) occupational therapy to restore independence in functional activities; (iv) psychological support for anxiety and depression; and (v) social work intervention to facilitate continuity of care. These multicomponent interventions can prevent, restore capacity, or reduce the severity of frailty-related complications after TAVI.

Achieving early discharge from hospital (≤5 days total length of stay (LOS)) in elective TAVI patients requires careful case selection and intensive perioperative management. Analysis of 512 transfemoral TAVI patients revealed that approximately 54.5% of elective patients could not achieve early discharge despite planned fast-track protocols due to comorbidities or procedural complications [31]. Frailty syndrome, renal impairment, new permanent pacemaker implantation, new bundle branch block, new atrial fibrillation, and life-threatening bleeding independently predicted failure to achieve ≤5-day LOS. These predictive factors should inform realistic patient expectations and resource planning while recognizing that extended hospitalization in frail patients may reflect appropriate medical management rather than procedural failure [31].

Futility Assessment and Shared Decision-Making Framework

Identifying patients unlikely to benefit from TAVI, those expected to experience no symptomatic improvement or functional recovery, remains a critical clinical challenge. Among 212 TAVI patients meeting guideline-defined futility criteria (either no symptomatic improvement or mortality at one year), lower baseline albumin and non-transfemoral approach independently predicted futility, alongside COPD, reduced eGFR, atrial fibrillation, low-flow, low-gradient stenosis, and low body mass index [6]. However, only 20% of patients meeting guideline-defined futility criteria perceived themselves as failing to benefit, with 80% versus 60% of non-futile patients reporting symptom remediation (p < 0.001). These data underscore that physician-determined futility based on objective parameters often diverges substantially from patient-perceived benefit, emphasizing the critical importance of integrating PROMs into decision-making frameworks [6].

Shared decision-making approaches incorporating pre-procedural baseline PROM and frailty assessment, followed by realistic discussion of anticipated functional recovery, substantially improve patient satisfaction, treatment concordance, and post-procedural engagement with rehabilitation [13]. Assessment of baseline frailty status through validated instruments (CFS, CGA components) helps clinicians and patients establish realistic expectations and identify potential for functional improvement versus likely persistent disability [3]. These conversations, informed by evidence-based prognostic models incorporating PROMs, create opportunities for explicit value alignment and alignment of treatment goals with individual patient priorities [8].

Reconciling clinical metrics with patient perspective: the futility paradox

Understanding the 80/60 Discordance

The landmark study by van Mourik et al. [6] examining 741 consecutive TAVI patients found that guideline-defined futility (defined as the combined endpoint of no symptomatic improvement OR mortality at one year) was present in 212 patients (28.6%). However, among patients with guideline-defined futility, 80% of those with symptomatic benefit estimated substantial symptom remediation compared to only 60% of those without benefit (p<0.001). Remarkably, the vast majority in both futile and non-futile groups stated they would undergo TAVI again, suggesting that physician-derived definitions of futility fundamentally misalign with patient-perceived value [6].

The France TAVI Registry (n=3,159) defined futility as death or significant quality of life degradation at one year and found one-third of patients met this criterion [32]. Yet even in this group, patients reported meaningful benefits. This is not an outlier finding-it reflects a systematic disconnect between how clinicians measure "success" and how patients experience it.

What This Discordance Reveals

Symptomatic improvement ≠ functional recovery: Patients report substantial symptom relief even when objective measures (NYHA class) show minimal improvement. A patient with baseline NYHA III dyspnea who improves to NYHA II with continued significant exercise limitation may perceive "50% symptom relief." This subjective improvement has genuine quality-of-life value even if clinical criteria would label them as non-responders.

Longevity ≠ quality of life priority: The original futility definition emphasizes mortality reduction. However, frail elderly patients frequently prioritize symptom relief and functional independence over maximal longevity. A patient with a one-year mortality risk of 30% but who achieves freedom from exertional syncope may rationally choose TAVI despite this risk, a choice that appears "futile" by clinical metrics but reflects authentic patient values.

Baseline expectations matter: Patients with severe frailty and low baseline function often have lower expectations for complete functional restoration. Maintaining functional status (as opposed to continued decline) represents a meaningful benefit. Long-term follow-up of 103 TAVI survivors at a median of seven years (5.4-9.8 years follow-up) found that 93.9% described improvement in quality of life despite 64.7% remaining in NYHA III-IV, suggesting that subjective well-being and functional class diverge significantly [33].

When Clinical Metrics Should Override Patient Preference (Genuine Futility)

The paradox does not mean clinical judgment is irrelevant. Several scenarios represent legitimate futility where intervention should be avoided. (i) Expected imminent mortality: patients with acute decompensated heart failure, sepsis, or active malignancy with life expectancy <6 weeks represent genuine futility independent of patient preferences [34]. (ii) Irreversible cognitive impairment preventing informed consent: patients unable to understand procedural risks and benefits cannot participate in shared decision-making [27]. (iii) Procedural contraindications: vascular anatomy precluding safe access or anatomical features making valve positioning impossible represent absolute contraindications, not futility definitions. (iv) Untreated modifiable conditions: patients with severe depression, active substance abuse, or inadequate medical optimization may not benefit from TAVI until these conditions improve. This represents a "bridge-to-decision" moment for optimization rather than definitive futility.

Framework for Clinical Decision-Making

The authors present a futility assessment framework that incorporates both objective risk and patient-centered values (Table 2).

**Table 2:** Framework for reconciling clinical metrics with patient perspective

Risk Category	Clinical Threshold	Baseline PROM	Patient Values	Physician Action	Reconciliation Approach	Expected 1-Yr Benefit
LOW RISK CANDIDATE	CFS ≤4, Albumin >35, eGFR >45, <15% 1-yr mortality	KCCQ >60, EQ-VAS >70	Values longevity + symptom relief; accepts procedural risk	Proceed with standard TAVI + routine rehabilitation	Clinical & patient goals aligned	>80% NYHA improvement ≥1 class
MODERATE-HIGH RISK	CFS 5, Albumin 30-35, eGFR 30-45, 15-30% mortality	KCCQ 40-60, EQ-VAS 50-70	Prioritizes symptom relief; accepts moderate procedural risk	Proceed + Enhanced risk counseling + Intensive monitoring	Goals largely aligned; detailed informed consent	60-80% NYHA improvement
HIGH RISK WITH MODIFIABLE FACTORS	CFS 5-6, reversible factors (malnutrition, depression, deconditioning)	KCCQ <40, EQ-VAS <50	Values independence & symptom relief; willing to optimize first	DEFER for 4-8 week PREHABILITATION TRIAL (nutrition, PT, mood treatment) then reassess	Maximize benefit by optimizing modifiable factors FIRST	50-70% improve post-prehab; highly variable otherwise
VERY HIGH RISK / GRAY ZONE	CFS 6-7, multiple comorbidities, 30-50% mortality, cognitively intact	KCCQ <30, EQ-VAS <40	Values symptom relief > longevity; may accept very high procedural risk	Multidisciplinary team discussion + Intensive monitoring if proceed + Early palliative care integration	Goals may diverge: clinical metrics suggest high risk, but patient values symptom relief—RESPECT PATIENT AUTONOMY if cognitively intact	40-60% experience any benefit; 20-40% fail to improve
GENUINE FUTILITY	>50% 1-yr mortality OR irreversible multi-organ failure OR acute life-threatening illness OR unable to consent	Cannot assess	Lacks decisional capacity OR explicitly declines	Transition to PALLIATIVE CARE: balloon valvuloplasty as temporizing measure, symptom management, goals-of-care counseling, hospice consideration	Clinical metrics OVERRIDE patient preference—futility is genuine	<20% meaningful benefit; 30-50%+ perioperative complications

Integrating PROMs Into Futility Assessment

Rather than using isolated clinical metrics, we recommend baseline PROM assessment as part of futility determination:

Patients with a baseline Kansas City Cardiomyopathy Questionnaire (KCCQ) score <40 and Clinical Frailty Scale (CFS) score ≥7 represent an extremely high-risk group but should not be automatically denied TAVI solely on these metrics [9].

Serial PROM assessment at 30 days and three months post-TAVI provides objective evidence of functional trajectory. Patients showing improvement in KCCQ despite initial perioperative complications represent delayed responders, not futility cases.

Patient-reported quality of life (EuroQol-5D-VAS) predicts long-term outcome better than some clinical parameters [11], supporting PROM integration into standard outcome assessment.

The Palliative Care Bridge

When TAVI is not appropriate, palliative care should not represent abandonment but rather optimization of patient-centered outcomes [34]. Balloon aortic valvuloplasty, sometimes dismissed as "bridge therapy," may provide meaningful symptom relief as an alternative to TAVI in extremely high-risk patients, allowing time for prehabilitation or clarification of patient goals.

Addressing the "Slippery Slope" Concern

A reasonable concern is that emphasizing the patient perspective might lead to inappropriate TAVI in genuinely futile cases. We address this by proposing that futility assessment requires triplet documentation: (i) Clinical futility: expected mortality or morbidity so severe that TAVI cannot reasonably alter disease trajectory. (ii) Procedural futility: technical factors (anatomy, vascular access) making TAVI unsafe or impossible. (iii) Decisional futility: patient lacks capacity to understand risks/benefits or explicitly declines intervention.

All three components must be present to justify denying TAVI. Patient-perceived benefit, by itself, does not override clinical or procedural futility-but patient values MUST inform clinical futility determination.

Practical implementation pathways for digital integration 

We present a realistic three-phase implementation strategy for digital integration:

Phase 1 (Immediate: 0-6 Months): Electronic Health Record Integration

Embedding validated frailty tools (CFS, SPPB, nutritional screening) into existing EHR workflows, creating standardized pre-TAVI assessment templates, and enabling automatic flagging of high-risk patients (CFS ≥5, albumin <35 g/dL, eGFR <30). The implementation barrier addressed is that most TAVI programs already have EHRs; this requires workflow redesign, not new technology.

Phase 2 (Near-term: 6-18 Months): Structured Reporting and Decision Support

Automated generation of risk reports combining CFS, nutritional markers, and functional assessments, integration of patient-reported outcome data at defined timepoints (baseline, 30 days, 1 year), and use of simple decision trees (not “AI,” but evidence-based algorithms) to flag patients at risk of functional decline. A real-world example is the GASS-TAVI model [2], which could be translated into a simple calculator requiring only CFS score, albumin, eGFR, and 6MWT time.

Phase 3 (Future: 18+ Months): Advanced Analytics

Machine learning models could potentially improve upon current risk prediction tools [35], alongside continuous remote monitoring via consumer wearables (activity trackers, smartwatches) to track actual functional recovery trajectories rather than relying on single baseline measurements. A realistic limitation is that current wearable data (step count, heart rate) do not differentiate between TAVI-related recovery and other factors, and more sophisticated biomarker integration would be needed.

## Conclusions

Patient-reported outcome measures and standardized frailty assessments represent essential components of contemporary TAVI patient evaluation, enabling truly patient-centered decision-making that extends beyond survival metrics to encompass quality of life, functional capacity, and treatment satisfaction. The Kansas City Cardiomyopathy Questionnaire, EuroQol instruments, and Short-Form health questionnaires provide robust quantification of symptom burden and functional limitation pre-TAVI and track meaningful changes post-procedure. Multiple validated frailty assessment tools, including the Clinical Frailty Scale, Comprehensive Geriatric Assessment components, Short Physical Performance Battery, and condition-specific frailty indices, demonstrate consistent associations with adverse TAVI outcomes and facilitate risk stratification beyond conventional cardiac risk scores.

Emerging TAVI-specific integrated risk models that incorporate patient-reported health status alongside objective comorbidity burden, nutritional markers, and physical performance demonstrate substantially superior predictive discrimination compared to traditional surgical risk calculators. Implementation of multidisciplinary TAVI programs centered on comprehensive geriatric assessment, coordinated nurse-led care, and personalized rehabilitation represents an evidence-based approach to optimizing outcomes in increasingly frail patient populations. Future research should prioritize: (a) prospective validation of TAVI-specific risk models incorporating PROMs and frailty measures in diverse populations; (b) randomized evaluation of preoperative prehabilitation and postoperative rehabilitation interventions tailored to frailty phenotype; and (c) integrating qualitative research into shared decision-making. Most critically, future TAVI programs should establish multidisciplinary geriatric heart teams (interventional cardiology, geriatric medicine, cardiac surgery, nursing, physical therapy, nutrition, and social work) as a standard organizational structure, recognizing that the value of frailty assessment lies in systematic team-based interpretation of multidimensional patient data integrated with explicit patient values clarification. This transforms assessment into a comprehensive optimization and shared decision-making framework feasible in any program regardless of resources.
